# Intercellular Extensions Are Induced by the Alphavirus Structural Proteins and Mediate Virus Transmission

**DOI:** 10.1371/journal.ppat.1006061

**Published:** 2016-12-15

**Authors:** Maria Guadalupe Martinez, Margaret Kielian

**Affiliations:** Department of Cell Biology, Albert Einstein College of Medicine, Bronx, New York, United States of America; Harvard Medical School, UNITED STATES

## Abstract

Alphaviruses are highly organized enveloped RNA viruses with an internal nucleocapsid surrounded by a membrane containing the E2 and E1 transmembrane proteins. Alphavirus budding takes place at the plasma membrane and requires the interaction of the cytoplasmic domain of E2 with the capsid protein. Here we used WT alphaviruses and Sindbis virus in which E2 was fused to a fluorescent protein to characterize virus exit from host cells. Our results show that alphavirus infection induced striking modifications of the host cell cytoskeleton and resulted in the formation of stable intercellular extensions that emanated exclusively from the infected cell. The intercellular extensions were long (> 10 μM), contained actin and tubulin, and formed flattened contacts with neighboring cells, but did not mediate membrane or cytoplasmic continuity between cells. Receptor down-regulation studies indicated that formation of stable extensions did not require the virus receptor, and that extensions promoted cell-to-cell virus transmission to receptor-depleted cells. Virus mutant experiments demonstrated that formation of extensions required the E2-capsid interaction but not active particle budding, while intercellular transmission of infection required the production of fusion-active virus particles. Protein expression studies showed that even in the absence of virus infection, the viral structural proteins alone induced intercellular extensions, and that these extensions were preferentially targeted to non-expressing cells. Together, our results identify a mechanism for alphavirus cell-to-cell transmission and define the key viral protein interactions that it requires.

## Author Summary

Alphaviruses are a group of small enveloped RNA viruses that include a number of important human pathogens such as Chikungunya virus and viruses that cause fatal encephalitis. Chikungunya virus emerged recently in a number of countries worldwide including the Americas, where it has caused major outbreaks. Vaccines and anti-viral strategies for these viruses are urgently needed, and basic information on the alphavirus infection pathway will help in targeting critical steps. Here we describe the changes in the alphavirus-infected cell that allow it to transmit virus to neighboring uninfected cells. Infected cells form long extensions that contact neighboring cells and mediate cell-to-cell virus transmission. This mechanism of virus transmission may help to shield virus from neutralization by host antibodies. Surprisingly, expression of the viral structural proteins alone induces these intercellular extensions, which preferentially target non-expressing cells. We used this system to define a critical interaction of the capsid and envelope protein that is required for formation of extensions.

## Introduction

Many viruses exploit and reorganize the host cell cytoskeleton during their entry and/or exit from the host cell [1–7]. The actin and microtubule-based systems can play key roles in virus endocytic uptake, uncoating, intracellular traffic, transport into the nucleus, and formation of virus replication centers. During exit, cytoskeletal proteins can promote transport of virus or viral components to the cell surface, polarized virus egress, and virus cell-to-cell transmission. Key intersections of the virus and host protein networks are involved in viral cytoskeleton use and remodeling. While the molecular mechanisms in many cases are not yet well-understood, they can differ dramatically among viruses and illuminate critical features that may be targets for anti-viral therapies.

Alphaviruses, members of a genus of the *Togaviridae* family, are small enveloped plus-strand RNA viruses that incude important human pathogens such as Chikungunya virus (CHIKV) and the encephalitic alphaviruses, as well as highly studied less pathogenic members such as Sindbis virus (SINV) and Semliki Forest virus (SFV) [8–10]. Alphaviruses contain a nucleocapsid core composed of the RNA genome enclosed in a lattice of 240 copies of the capsid protein (Cp). This core is surrounded by the viral envelope, a lipid bilayer containing the E2 and E1 transmembrane glycoproteins arranged in a lattice of 80 trimers of E2/E1 heterodimers. The E2 protein binds to host cell surface receptors and mediates endocytic uptake of virus, while E1 triggers virus membrane fusion in the low pH environment of the endosome. Alphaviruses replicate in the cytoplasm and bud from the plasma membrane (PM). Budding of the virus involves the one-to-one interaction of the cytoplasmic domain of E2 with a hydrophobic pocket on the capsid protein. Mutations in this critical region of E2 (e.g., SINV E2 Y400K or SFV E2 Y399R) block E2-Cp interaction, nucleocapsid localization at the PM, and virus budding [11]. Particle production is also dependent on the dimeric interactions of E2 and E1, and is enhanced by the small 6K membrane protein. The highly organized alphavirus particles exclude host membrane proteins during their budding [10, 12].

Early electron microscopy studies indicated that alphavirus budding occurs at a variety of sites on the PM including peripheral regions, patches, and short processes [13–15]. To follow the alphavirus assembly and budding pathway, we and others developed SINV constructs containing various fluorescent proteins at the N-terminus of the E2 membrane protein [12, 16, 17]. We found that green fluorescent protein (GFP)- or mCherry-labeled viruses are functional for assembly and infection, with equivalent results for both labels [12]. The structures and budding patterns of the labeled viruses resemble those of WT SINV, and the labeled E2 proteins accurately reflect the intracellular distribution of the WT E2 protein [12, 16]. Infection was found to induce a dramatic remodeling of the host cell, including formation of both the previously described budding structures and long (>10 μm) cellular processes that can release alphavirus-sized particles [12, 16]. Alphavirus infection has been considered a classic example of released progeny virus infecting a distant new host cell. While cell-free alphavirus particles indeed efficiently infect host cells, cell-to-cell transmission has also been postulated based on relative resistance to antibody neutralization [18]. This mode of virus transmission can involve virological synapses closely opposing infected and target cells, or cellular connections via nanotube or filopodia-like extensions [1, 6].

Here we have addressed the properties of the extensions produced during alphavirus infection, their function in mediating cell-to-cell virus transmission, and the viral protein requirements for their induction. We found that long tubulin and actin-positive extensions emanated exclusively from infected cells and directly and stably contacted neighboring cells. Formation of these extensions was conserved among alphaviruses, occurred in a variety of cell lines including primary cultures, and mediated intercellular virus transmission. Extensions were induced by the expression of the alphavirus structural proteins alone, in the absence of virus infection, through a mechanism that required the E2-Cp interaction and preferentially targeted non-expressing cells.

## Results

### Alphavirus infection induces the formation of actin-positive intercellular extensions

**Fig 1:**
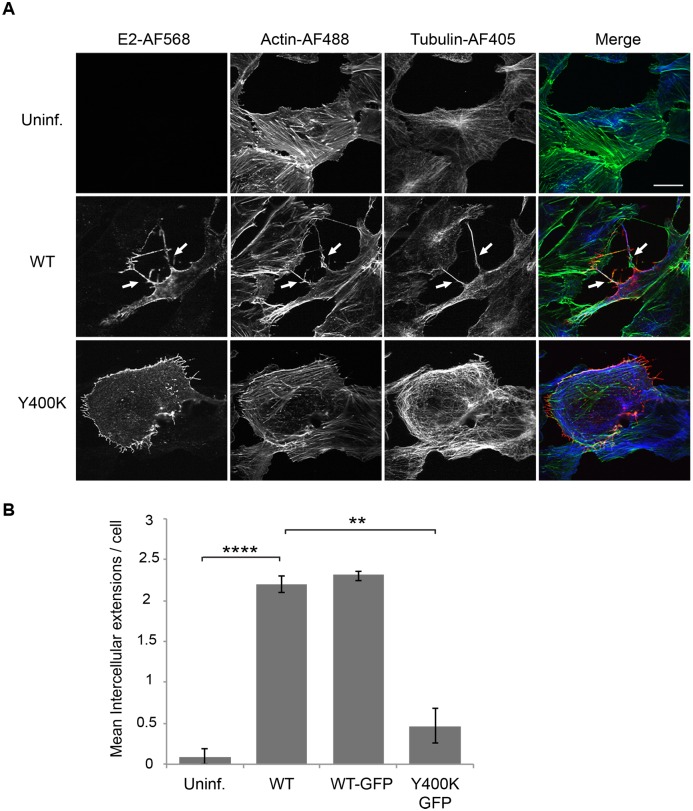
Alphavirus infection induces actin-positive intercellular extensions. (A) Vero cells were mock-transfected (Uninf.) or transfected with WT SINV or Y400K RNA, incubated at 37°C for 8 h, and fixed. Cells were permeabilized and stained with antibodies to detect the viral E2 envelope protein (red) and α-tubulin (blue), and with phalloidin to detect F-actin (green). Cells were imaged by confocal microscopy. Images from one optical section are shown and are representative of three independent experiments, which are quantitated in (B). Arrows indicate two examples of intercellular extensions; note that each is positive for all three markers and is in contact with a neighboring cell. Bar = 20 μm. (B) The number of intercellular extensions per infected cell (n = 10) was quantitated based on their positive staining for E2, actin and tubulin and their contact with a neighboring cell (see methods). Graph in B shows the mean and standard deviation of three independent experiments, with 10 cells quantitated in each sample including the uninfected cells. ** P<0.01, ****P<0.0001.

SINV infection of Vero cells resulted in the formation of long (> 10 μM) filopodia-like extensions that stained positively for both actin and tubulin (Fig 1A). As in our previous work [12], we observed that SINV infection also induced the formation of short (~2–4 μM) extensions (Fig 1A). The long actin/tubulin-positive extensions were often branched, and their overall length varied between ~10–60 μm. They were detected starting at ~6–7 h post-infection. We also tested a SINV mutant in which mutation of a critical tyrosine in the E2 internal domain impairs E2-Cp interaction and inhibits virus budding (SINV E2 Y400K) [11]. While mutant-infected cells showed abundant short extensions, they showed significantly reduced levels of the long-actin/tubulin positive extensions (Fig 1A, lower panel).

Confocal microscopy images showed that many of the longer extensions induced by the WT virus extended to and were in apparent physical contact with neighboring cells. We therefore quantitated the number of such “contacting” extensions in the WT, WT-GFP, or Y400K-GFP infected cells (Fig 1B). WT or WT-GFP infection induced a significant increase in intercellular extensions, while the mutant-infected cells showed no significant increase. Thus, infection of Vero cells with budding-competent SINV induced the formation of long actin/tubulin positive extensions that contact neighboring cells. We will refer to these specific structures as “intercellular extensions”.

### Properties of intercellular extensions

**Fig 2:**
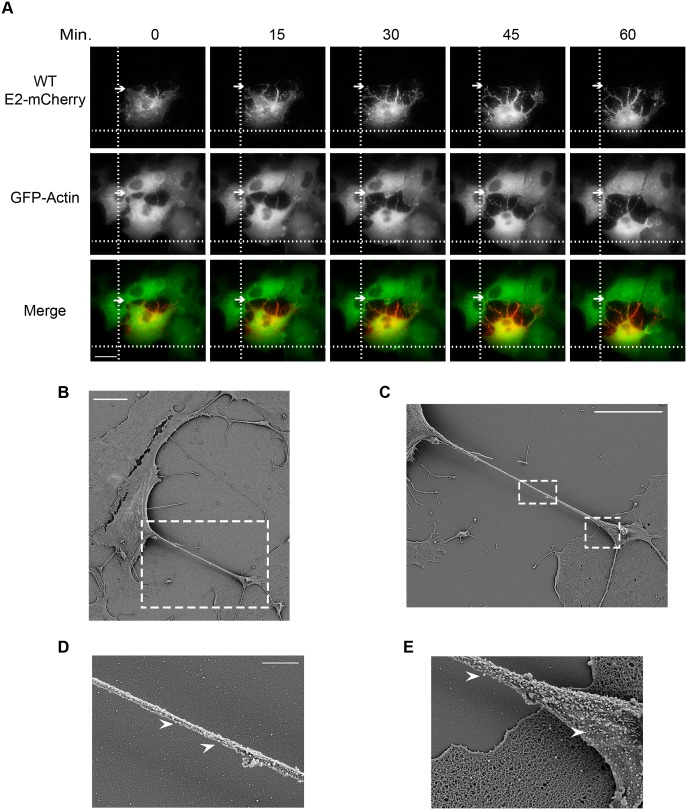
Intercellular extensions are generated by stable cell-cell contacts and migration of infected cells, and contain budding virus particles. (A) Vero cells stably expressing GFP-actin were infected with WT-mCherry SINV and incubated for 7 h at 37°C. The cells were then imaged using the TIRF microscope in the wide-field mode and both the 561-nm and 488-nm lasers. Images were acquired every 10 s for 60 min (see S1 Movie). Arrows indicate stable contacts established between an infected cell and a neighboring cell, and marker lines are used to follow cell position during migration. Bar = 20 μm. (B-E) Vero cells were infected with WT SINV, incubated at 37°C for 9 h, fixed, processed for SEM, and imaged using a Zeiss Supra 40 field emission SEM. (B) A representative image of infected cells. Bar = 10 μm. (C) SEM image of the region indicated by the dashed white box in panel B. Bar = 10 μm. (D and E) SEM images of the regions indicated by the dashed white boxes in panel C. Arrowheads indicate virus-sized structures. Bar = 1 μm.

To characterize the formation of intercellular extensions, we performed live cell imaging/time-lapse microscopy on Vero cells stably expressing GFP-actin and infected with SINV WT E2-mCherry (Fig 2A, see also S1 Movie). We observed that the intercellular extensions originated exclusively from infected (mCherry-positive) cells. When an infected cell contacted a neighboring cell, it frequently established very stable interactions that remained intact during the subsequent migration of the cell in the opposite direction. This movement away from the neighboring cell resulted in the elongation of the intercellular extensions, while the original contact site was stably maintained. Thus, the intercellular extensions did not result from passive retraction of the infected cell.

We then used scanning electron microscopy (SEM) to characterize the extensions. Representative SEM images of Vero cells infected with WT virus showed long extensions emanating from infected cells, which were readily identified by the presence of abundant virus-sized particles on their cell surfaces. The region of the extension mediating contact between the infected cell and a neighboring cell usually formed an extensive flattened tip that contained a high density of virus-sized particles at its surface (Fig 2B, 2C and 2E). SEM images also showed virus-sized particles present along the length of the extensions (Fig 2D). Immunofluorescence analysis showed that the extensions contain the viral envelope and capsid proteins, in keeping with the presence of virus particles in these extensions (S1 Fig).

**Fig 3:**
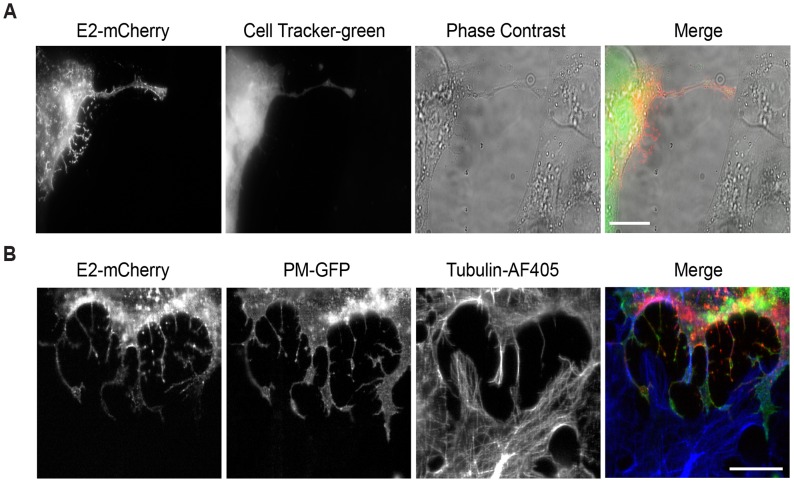
Intercellular extensions do not transfer cytosolic or PM markers. (A) Vero cells were transfected with SINV WT-mCherry RNA (t = 0) and incubated for 6.5 h at 37°C. Cells were labeled for 30 min with CellTracker Green and then uninfected Vero cells were added and the co-cultures were incubated for 2 h at 37°C. Cells were then fixed and imaged by epifluorescence microscopy. Images are representative of three independent experiments, evaluating 10 cell pairs/experiment. Bar = 20 μm. (B) Vero cells expressing the PM-GFP marker were transfected with SINV WT-mCherry RNA and incubated at 37°C for 7 h. Uninfected Vero cells were then added and the co-cultures were incubated for 2 h at 37°C. Cells were fixed, stained with antibodies to detect α-tubulin, and imaged by confocal microscopy. Images from one optical section are shown, and are representative of three independent experiments, 10 cell pairs/experiment. Bar = 20 μm.

An important property of intercellular extensions generated between two cells is the extent of the continuity between them. Once contact is established, the membranes of the cells can fuse, generating “open-ended” connections detectable by the continuity of the plasma membranes and possibly the cytoplasms of the two cells [6, 19]. We tested whether the soluble cytosolic dye Cell Tracker Green could be delivered from labeled infected cells to unlabeled target cells. We also tested if a freely diffusing plasma membrane marker (PM-GFP) [12] on the infected cell could be delivered to unlabeled target cells. In each case, while frequent extensions between labeled infected cells and unlabeled target cells were generated, no transfer of the soluble cytoplasmic marker or the freely diffusing PM-GFP protein was observed (Fig 3). These results suggest that the membranes at the contact site of the infected cell and target cell do not fuse.

### Formation of intercellular extensions by various alphaviruses and cells

**Fig 4:**
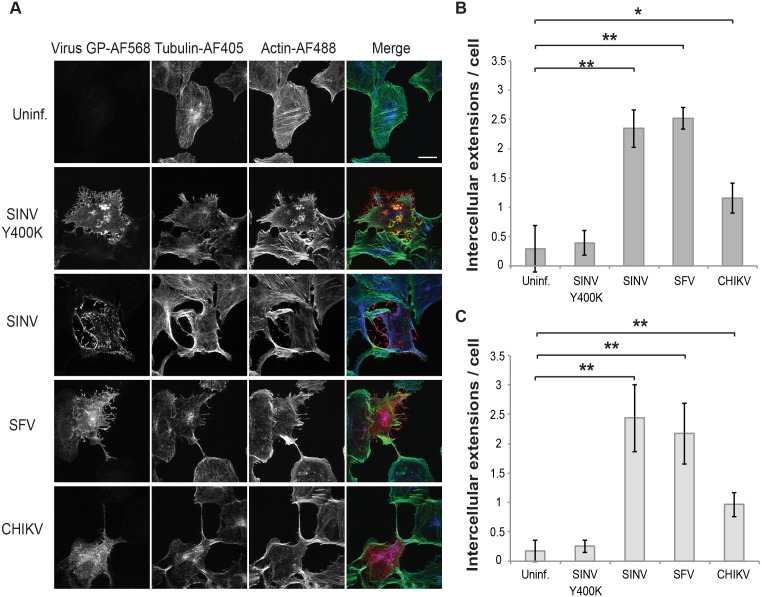
Intercellular extensions are induced by various alphaviruses in Vero and primary human cells. (A) Vero cells were mock-infected (Uninf.), transfected with SINV Y400K RNA, or infected with WT SINV, SFV or CHIKV (MOI 20, 10, 10, respectively). Cells were then incubated at 37°C for 11 h, fixed, permeabilized, and stained with antibodies to detect viral envelope proteins (virus GP) and α-tubulin, and with phalloidin to detect F-actin. Cells were imaged by confocal microscopy. Images from one optical section are shown and are representative of three independent experiments. Bar = 20 μm. (B) Quantitation of the number of extensions in Vero cells from experiments as described in (A). Intercellular extensions emanating from infected cells were identified based on positive staining for GP, actin and tubulin and contact with a neighboring cell (see methods). (C) HUVECs were infected and stained as in (A) and imaged and quantitated as in (B). Graphs in B and C show the mean and standard deviation of three independent experiments, with 10 cells quantitated in each sample including the uninfected cells. * P<0.05, ** P<0.01,*** P<0.001.

To determine if intercellular extensions were also induced during infection by other alphaviruses, Vero cells were infected with SINV, SFV, CHIKV, or the non-budding SINV mutant Y400K. The SFV and CHIKV-infected cells generated intercellular extensions similar to those seen in SINV-infected cells, while cells infected with the non-budding Y400K mutant generated few intercellular extensions (Fig 4A and 4B). In addition, efficient formation of intercellular extensions was also observed in Vero cells infected with clinical isolates of SINV and SFV.

As Vero cells are a transformed cell line, we tested whether intercellular extensions were induced during alphavirus infection of primary cells. We infected primary human umbilical vein endothelial cells (HUVEC) with several alphaviruses and quantitated the presence of intercellular extensions (Fig 4C, S2 Fig). Similar to Vero cells, alphavirus infection of HUVEC induced the formation of intercellular extensions, and formation was reduced for the non-budding Y400K mutant. Similar formation of extensions was observed following SINV infection of U2-OS, CHO, and 3T3 cells. Interestingly, we did not observe intercellular extensions in SFV-infected U2-OS cells or in SFV or CHIKV-infected CHO cells. This difference is currently under investigation.

### SINV cell-to-cell transmission

**Fig 5:**
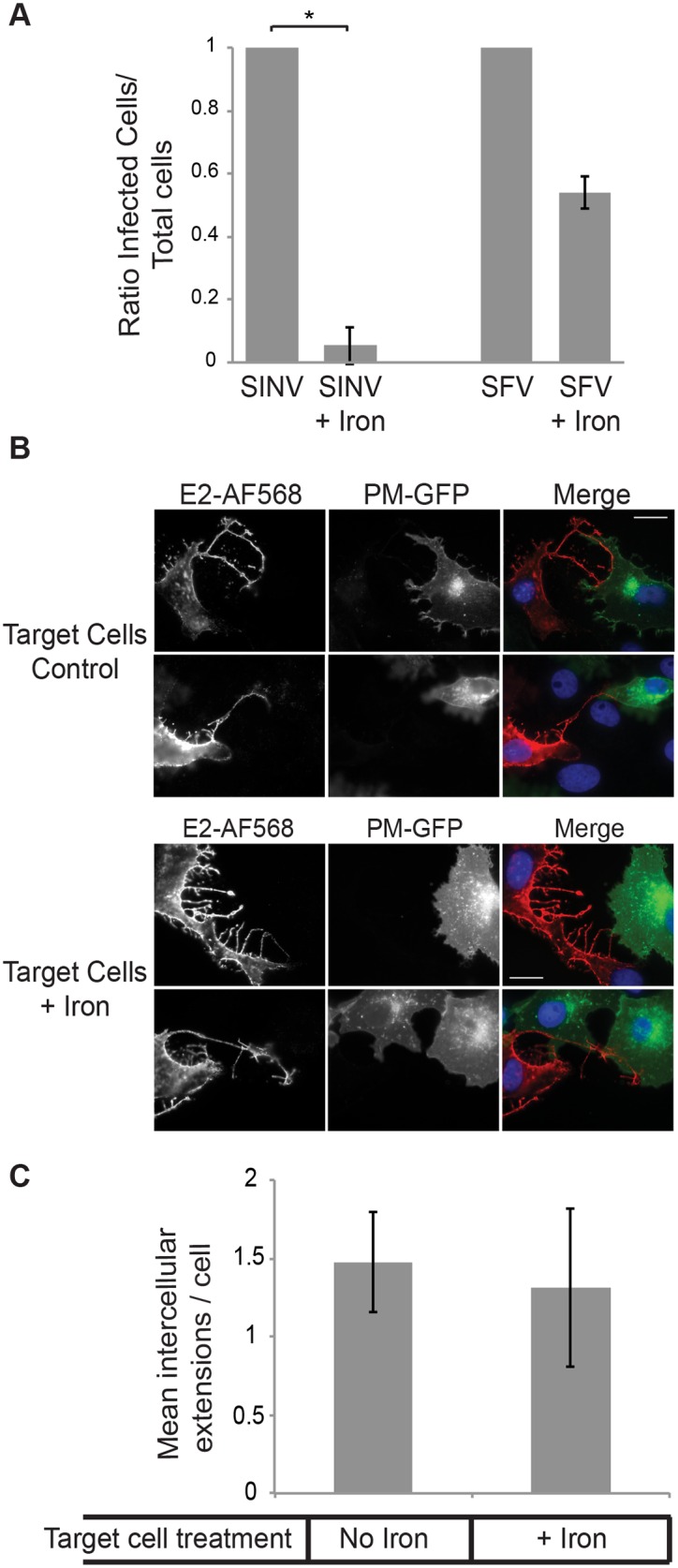
Formation of intercellular extensions and virus cell-cell transmission are independent of NRAMP2. (A) Effect of NRAMP downregulation on free virus infection. Vero cells were cultured for 3 days in control media or media containing 200 μg/ml ammonium iron citrate to down-regulate the SINV receptor NRAMP2. Cells were infected with SINV or SFV (MOI = 5) for 2 h at 37°C. 20 mM NH_4_Cl was then added to the medium to prevent secondary infection. Cells were fixed at 24 h post-infection and the ratio of infected to total cells quantitated by staining with antibody to the SINV or SFV E2 protein. The graph represents the mean and standard deviation of three independent experiments, with infection normalized to that of control cells (which was set to 1). * P<0.05. (B) Effect of NRAMP downregulation on formation of intercellular extensions. Vero cells were infected with SINV (MOI = 5) for 5 h at 37°C. Target Vero cells stably expressing the PM-GFP marker were pretreated as in (A) to downregulate NRAMP2, and then plated onto the infected cells at an approximate ratio of 1:1. The co-cultures were incubated for 3 h at 37°C in the continued presence of iron as indicated, fixed, permeabilized, and stained with antibody to SINV E2. Fluorescence microscopy images were acquired with the same exposure time, and images are representative of the results of 3 independent experiments. Bar = 20 μM. (C) Quantitation of the number of extensions in Vero cells from experiments as described in (B). Intercellular extensions emanating from infected cells were identified based on positive staining for the SINV E2 protein and contact with a PM-GFP expressing target cell, as described in the methods. The graph in (C) represents the mean and standard deviation of three independent experiments.

The generation of stable intercellular extensions by infected cells suggested that the formation and/or stabilization of these specialized processes might be mediated by interactions with the alphavirus receptor and could promote alphavirus transmission. To test these ideas, we set up a co-culture system in which the expression of the SINV receptor, natural resistance-associated macrophage protein 2 (NRAMP2) [20], was down regulated in target cells. NRAMP2 is a metal ion transporter sensitive to intracellular iron concentrations; culture in the presence of elevated levels of iron leads to reduced levels of NRAMP2 [20]. SINV infection of Vero cells pretreated with iron was reduced by >90% compared to control cells (Fig 5A). In contrast, infection by SFV, which does not use NRAMP2 as a receptor, was not significantly decreased by iron pretreatment. We used this system to determine if interaction between E2 on the infected cells and NRAMP2 on target cells promoted the formation of stable intercellular extensions. “Producer” Vero cells were infected for 5h with SINV. “Target” Vero cells stably expressing the PM-GFP marker were precultured under control or high iron conditions, and plated onto the infected cells. The cells were fixed after 3 hours of co-culture. As shown in Fig 5B and 5C, comparable numbers of intercellular extensions were formed between producer and target cells whether or not NRAMP2 was down-regulated. Thus E2-NRAMP2 interaction does not appear responsible for the stable interaction between cells. Cells deficient in glycosaminoglycans [21], which are known to promote alphavirus attachment [22], also formed intercellular extensions (S3 Fig). We also evaluated if stabilization might involve “frustrated phagocytosis” as observed for retrovirus-induced extensions [6]. We did not observe accumulation of the endocytic markers dynamin, clathrin, or caveolin at the contact sites of producer and target cells (S4 Fig).

Since downregulation of the NRAMP2 receptor greatly inhibited infection by free SINV (Fig 5A), we used this system to test if the extensions were able to mediate cell-to-cell virus transmission. Target cells expressing PM-GFP were pretreated with or without iron, co-cultured for 19 h with SINV- or SFV-infected producer cells, and infection of the GFP-positive target cells quantitated by immunostaining. Unlike the ~90% block observed for free SINV infection of NRAMP2 down-regulated cells, infection mediated by co-culture with SINV-infected cells was not significantly decreased, and was comparable to that observed with SFV co-cultures (S5 Fig). This result suggests that under these conditions the target cells are predominantly infected by cell-to-cell transmission.

**Fig 6:**
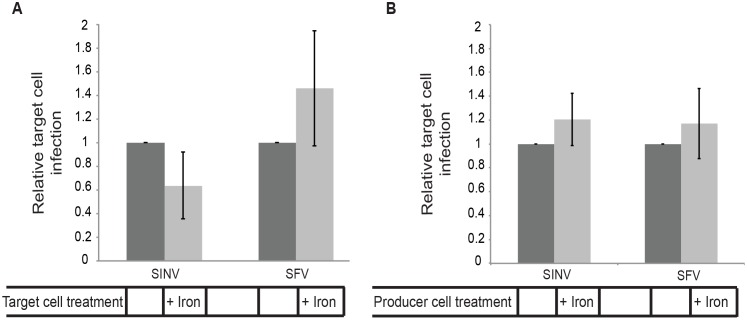
SINV cell-cell transmission is independent of NRAMP in target or producer cells. (A) Down-regulation of target cell NRAMP. Vero cells were transfected with WT SINV or SFV RNA and incubated at 37°C for 5 h (producer cells). Target Vero cells stably expressing the PM-GFP marker were cultured for 3 days in control media or media containing 200 μg/ml ammonium iron citrate to down-regulate the SINV receptor NRAMP2, and then plated onto the infected cells at an approximate ratio of 1:1 and the co-cultures incubated for 19 h at 37°C in the continued presence of iron as indicated. The % infected target cells was quantitated by staining with antibody to the SINV or SFV E2 protein. The graph represents the mean and standard deviation of three independent experiments, with infection normalized to that of control target cells (which was set to 1). (B) Down-regulation of producer cell NRAMP. Vero cells were pretreated as in Fig 6A to downregulate NRAMP2, transfected with WT SINV or SFV RNA, and incubated at 37°C for 5 h (producer cells). Uninfected Vero cells stably expressing PM-GFP (target cells) were then plated onto the infected cells, and the co-cultures were incubated for 19 h at 37°C in the continued presence of iron as indicated. Infection of target cells was quantitated as in Fig 6A. The graphs in A and B represent the mean and standard deviation of three independent experiments.

Since the producer cell cultures were initially infected by adding free virus, it was possible that infection of the co-cultured target cells was mediated by residual virus bound to the culture dish. We therefore infected the producer cells by transfection with SINV or SFV viral RNA and determined their ability to mediate infection of co-cultured target cells with or without NRAMP2 down-regulation. As shown in Fig 6A, target cells were efficiently infected by viral RNA-transfected producer cells, even when the NRAMP2 receptor was down-regulated. Transfection of producer cells with RNA from the non-budding SINV E2 Y400K mutant did not produce infection of target cells, confirming that target cells were infected by released virus rather than residual RNA from the transfection (S6 Fig). Together the results in Fig 6A and S5 Fig indicate that NRAMP-depleted target cells were efficiently infected by producer cells even when compared with control target cells, where virus infection can be mediated by both released “free” virus and by cell-cell transmission.

As an additional approach, we analyzed cell-to-cell spread of SINV in the presence of neutralizing antibodies to inhibit infection by virus in the medium. Under these conditions, intercellular extensions were formed from infected cells, and microplaques indicative of virus intercellular transmission were observed (S7 Fig). Using iron down-regulation, we also tested the requirement for receptor in the producer cell to mediate virus transmission. Producer cells were pretreated with iron, transfected with viral RNA, and infection of co-cultured target cells evaluated (Fig 6B). There was no requirement for receptor in the producer cells for cell-to-cell transmission.

### Intercellular transmission occurs via fusion-active virus particles

**Fig 7:**
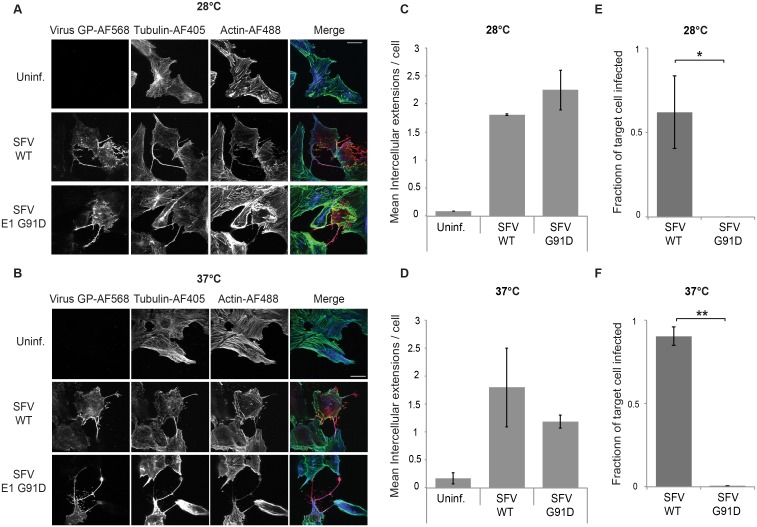
Formation of intercellular extensions does not require virus budding. Vero cells were mock-transfected (Uninf.) or transfected with SFV WT or G91D mutant RNA, incubated at the permissive temperature (28°C) overnight (A) or at the non-permissive temperature (37°C) for 8 h (B), and fixed. Note that different times of incubation at these two temperatures were used to allow virus replication and extension formation. Cells were permeabilized and immunostained to detect the virus glycoproteins (GP) or α-tubulin, and stained with phalloidin-Alexa488 to detect F-actin. Cells were imaged by confocal microscopy. Images from one optical section are shown and are representative of the images from three independent experiments. Bar = 20 μm. (C,D) The number of intercellular extensions per infected cell (n = 10) was quantitated based on their positive staining for GP, actin and tubulin and their contact with a neighboring cell. (E,F) Vero cells were transfected with WT or G91D SFV RNA and incubated at 37°C for 2h (producer cells). At 4 h post-transfection Vero target cells stably expressing the PM-GFP marker were plated onto the infected cells at an approximate ratio of 1:1 and the co-cultures incubated overnight at 37 or 28°C. Cells were then fixed, permeabilized, and immunostained to detect the viral glycoproteins. Epifluorescence microscopy was used to acquire 5 images using the 20X objective. The number of infected PM-GFP-positive target cells was quantitated and expressed as a fraction of the total number of target cells. The graphs in C-F represent the mean and standard deviation of three independent experiments. * P<0.05, ** P<0.01.

Our data to this point indicated that actin/tubulin positive extensions were induced by infection with WT alphaviruses but not with the non-budding Y400K mutant. Thus alphavirus budding per se could provide the signal to induce formation of intercellular extensions that can mediate virus transmission. To test for the role of virus budding, we took advantage of a previously characterized SFV mutant, E1 G91D [23, 24]. The E1 G91D mutation blocks fusion and infection, but also causes a temperature-sensitive budding phenotype. G91D virus buds efficiently at 28°C. However, G91D budding is greatly decreased at 37°C, although E2, E1 and nucleocapsids are efficiently delivered to and localized at the plasma membrane. Thus, this mutant differs from the E2 Y400K mutant, in which nucleocapsid is not recruited to the plasma membrane due to the lack of E2 interaction [11]. To test the effect of the G91D mutation on the formation of extensions, Vero cells were transfected with WT or G91D RNA, cultured at 37°C or 28°C, fixed, and analyzed by immunofluorescence (Fig 7). Both WT and mutant virus induced the formation of actin and tubulin-positive intercellular extensions (Fig 7A and 7B), and quantitation showed that there was no significant difference between induction by WT and G91D at either temperature (Fig 7C and 7D), indicating that budding per se is not required to induce extensions as long as the viral envelope proteins and nucleocapsid are recruited to the plasma membrane. However, co-culture experiments showed that the fusion-negative G91D virus did not mediate intercellular transmission even under budding-permissive 28°C incubation conditions (Fig 7E and 7F).

**Fig 8:**
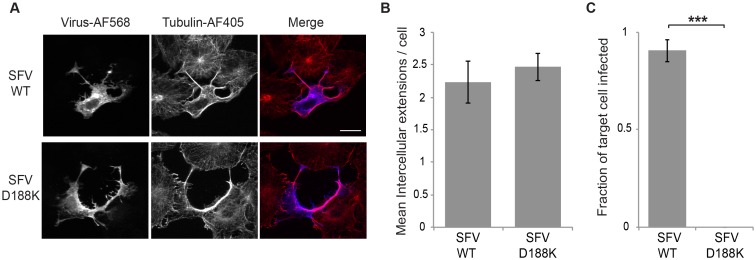
Production of fusion-active virus is required for cell-to-cell transmission. (A) Vero cells were transfected with SFV WT or D188K mutant RNA, incubated at 37°C and fixed at 8 hours post transfection. Cells were permeabilized and immunostained to detect the virus E2 glycoprotein or α-tubulin, and imaged by confocal microscopy. Images from one optical section are shown and are representative of the images from three independent experiments. Bar = 20 μm. (B) The number of intercellular extensions per infected cell (n = 10) was quantitated based on their positive staining for E2 and tubulin and their contact with a neighboring cell. (C) Vero cells were transfected with WT or D188K SFV RNA and incubated at 37°C for 2 h (producer cells). At 4 hours post transfection Vero target cells stably expressing the PM-GFP marker were plated onto the producer cells at an approximate ratio of 1:1 and the co-cultures incubated at 37°C overnight. Cells were then fixed, permeabilized, and immunostained to detect the viral glycoproteins. Epifluorescence microscopy was used to acquire 5 images using the 20X objective. The number of infected PM-GFP-positive target cells was quantitated and expressed as a fraction of the total number of target cells. The graphs in B and C represent the mean and standard deviation of three independent experiments. *** P<0.001.

To confirm that cell-to-cell transmission required production of fusion-active virus, we also tested cells infected with the fusion-inactive SFV mutant E1 D188K [25]. This mutant assembles and produces virus particles with comparable efficiency to WT virus, but the virus is fusion-inactive and non-infectious. Vero cells were transfected with WT or D188K RNA, cultured at 37°C, and intercellular extensions quantitated. Both WT and the D188K mutant efficiently and comparably induced intercellular extensions (Fig 8A and 8B). However, when tested in co-culture experiments, WT-transfected cells mediated infection of neighboring GFP-labeled target cells, while no intercellular transmission of the G91D mutant was observed (Fig 8C). Thus, alphavirus cell-to-cell transmission required production of fusion-active virus particles, demonstrating that it was not mediated by cell-to-cell transfer of viral RNA or replication complexes.

### Structural proteins alone mediate the formation and targeting of intercellular extensions

**Fig 9:**
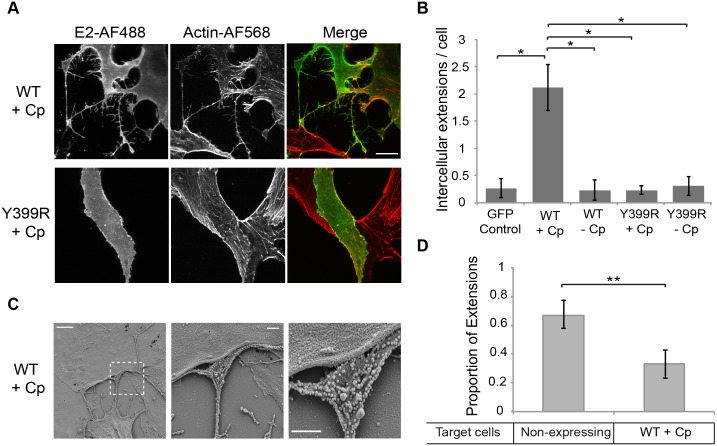
The alphavirus structural proteins induce the formation of intercellular extensions that preferentially target non-expressing cells. (A) Vero cells were transfected with plasmids encoding the SFV structural proteins with or without capsid and with or without the E2 Y399R mutation. At 24 h post-transfection the cells were fixed, permeabilized, immunostained for the SFV E2 protein and stained with phalloidin-Alexa568 to detect F-actin. Images were acquired by confocal microscopy and are representative of three independent experiments. Images from one optical section are shown. Bar = 20 μm. (B) Using the conditions described in (A), the number of intercellular extensions per E2-expressing cell was quantitated based on positive staining for actin and contact with a neighboring cell. The graph represents the mean and standard deviation of three independent experiments. *P<0.05. (C) Vero cells were transfected with a plasmid encoding the SFV structural proteins including capsid, incubated at 37°C for 24 h, fixed, processed for SEM and imaged using a Zeiss Supra 40 field emission SEM. Shown are representative SEM images. Left panel: Bar = 5 μm. Middle panel: Bar = 1 μm. Right panel: 2X zoom of middle panel, bar = 1μm. (D) Vero cells were transfected with a plasmid encoding the SFV structural proteins including capsid, incubated at 37°C for 24 h, fixed, permeabilized, and immunostained for the SFV E2 protein and α-tubulin. The intercellular extensions per expressing cell were quantitated based on their positive staining for tubulin and their contact with another cell, and scored based on expression of SFV E2 in the target cell. The graph represents the mean and standard deviation of three independent experiments. ** P<0.01.

To dissect the mechanism of formation of intercellular extensions, we transiently expressed the SFV structural proteins in Vero cells using expression vectors. A similar CHIKV protein expression system was previously shown to produce virus-like particles [26]. Vectors were constructed to express the SFV structural proteins with or without the Cp and with or without the E2 Y399R mutation that blocks virus budding. Vero cells transfected with a vector encoding the WT SFV structural proteins (Cp-p62-6K-E1) produced actin/tubulin positive intercellular extensions similar to those induced in virus-infected cells (Fig 9A upper panels). Extensions were not observed in cells expressing the SFV structural proteins plus the E2 Y399R mutation or minus the Cp, or in cells transfected with a control plasmid (Fig 9A and 9B).

Thus, expression and interaction of E2 and Cp appeared to trigger the formation of long, actin- and tubulin-containing intercellular extensions. SEM analysis of cells expressing the WT structural proteins showed extensions that were morphologically similar to those induced by virus infection (Fig 9C). Abundant virus-like particles were observed along the extensions and were enriched on the flattened surface that contacted the neighboring cell (Fig 9C), similar to the virus-sized particles that were observed for extensions in virus-infected cells (Fig 2).

Our results showed that during virus infection intercellular extensions emanated exclusively from infected cells and mediated cell-to-cell transmission. These results raised the question of whether intercellular extensions preferentially targeted non-infected cells. This question could be more cleanly addressed in cells expressing the virus structural proteins, avoiding the possibility of early, non-detectable levels of infection. Vero cells were transfected with a vector expressing the SFV structural proteins and fixed after 24h. Expressing cells were detected with antibody to the E2 protein and total cells were detected with antibody against tubulin. Intercellular extensions were quantitated and their contact with E2-positive vs. E2-negative cells was determined (Fig 9D). The results showed that formation of intercellular extensions from expressing cells is indeed strongly, although not exclusively, targeted to non-expressing cells.

## Discussion

Our results showed that alphavirus infection induced dramatic alterations in the cell cytoskeleton, resulting in the formation of long actin- and microtubule-containing intercellular extensions. Formation of these intercellular extensions was conserved among alphaviruses and occurred in a variety of cell lines including primary cell lines. Live cell imaging showed that once intercellular contact was established, the connections tended to be very stable even in the face of considerable movement of both cells. The intercellular extensions enhanced the cell-to-cell spread of virus infection in receptor down-regulated cells. Expression of just the viral structural proteins alone, in the absence of virus infection and the non-structural proteins, induced similar cytoskeletal remodeling and extensions, which preferentially targeted non-expressing cells.

The intercellular extensions we describe here have distinct biochemical and morphological characteristics. While the nomenclature for the wide variety of intercellular connections currently described in the literature is not standardized and remains confusing [6, 27], it is useful to point out specific features of the alphavirus-induced extensions for comparison. We observed that alphavirus-induced extensions contained both F-actin and microtubules, were branched and had a relatively large diameter, and did not extensively contact the substratum. The site contacting the target cell frequently showed a flattenned tip. Unlike nanotubes, these extensions did not appear to directly connect the cytoplasms of the two contacting cells, since we did not observe transfer of the plasma membrane marker PM-GFP or labeled E2, or the cytoplasmic dye CellTracker-green. While the extensions appear to promote alphavirus cell-to-cell transmission, transmission required production of fusion-active virus particles, rather than being mediated by transfer of the viral replication complex or viral RNA. Nanotubes contain F-actin and can contain microtubules, and do not contact the substratum, but in contrast to the intercellular extensions we describe, nanotubes mediate direct cytoplasmic connections and can transfer markers including various organelles (early endosomes, endoplasmic reticulum or Golgi-derived vesicles, lysosomes, mitochondria) and plasma membrane components [19, 27, 28]. Filopodia contain actin filaments and mediate long distance intercellular connections without cytoplasmic continuity, but show extensive substratum contacts [1]. Together the properties of the alphavirus-induced extensions point out both the ability of the virus to co-opt the cellular cytoskeleton and its selective use of this cellular machinery.

Our results indicated that the intercellular extensions are positive for alphavirus E2 protein along their length and are formed by initial cell contacts or protrusions emanating from a virus-infected cell, rather than by processes generated from an uninfected cell. The directionality of the extensions generated from the alphavirus-infected cell is thus the opposite of those described in retrovirus cell-to-cell transmission [29, 30]. For retroviruses, receptor-positive filopodia emanate from the uninfected cell and are stabilized by binding to envelope-positive infected cells and frustrated phagocytosis. The resulting stable bridges mediate the transfer of budded retrovirus particles from the infected cell to the uninfected cell by retrograde actin flow. Such retrograde flow is clearly not involved in alphavirus cell-to-cell transmission along extensions, since it would transport budded virus back to the infected cell. Instead, we favor a working model in which actin mediates the formation of the extensions and localized enrichment of budding virus at the close sites of cell-cell contact enhances virus transmission, even in receptor down-regulated cells. The intracellular transport of viral components to these contact sites could be mediated by the actin machinery, and/or by the microtubules within the extensions. Interestingly, a screen of human trafficking genes and subsequent analyses indicated that actin and proteins involved in actin-remodeling are involved in mediating transport of the alphavirus E2 protein to the cell surface [31].

We found that alphavirus-induced extensions were preferentially targeted to cells not expressing viral proteins, and that once contacts were formed the two cells displayed a very stable interaction that remained intact during their subsequent migration. Our results suggest that the formation and stabilization of the intercellular extensions did not require the virus receptor, heparan sulfate attachment factors, or the process of frustrated phagocytosis. We speculate that infected cells randomly contact neighboring cells, and that stabilization of cell-cell contacts is favored for uninfected targets, producing the preferential targeting of extensions to non-expressing cells that we observe. The molecular mechanism of preferential targeting/stabilization remains to be defined, and reflects important differences in the intercellular transmission processes among major viral pathogens.

While alphavirus budding per se was not required, the expression of the structural proteins and the interaction of E2 and Cp was essential to induce cytoskeletal rearrangements and formation of intercellular extensions. The requirement of the E2-Cp interaction for particle budding is conserved among different alphaviruses, and comparable mutations in the E2 cytosolic tail inhibit their budding [11, 12]. How might this conserved E2-Cp interaction signal to the cytoskeletal network to promote formation of actin and microtubule-containing branched cellular extensions? The alphaherpesvirus also induce actin and MT-positive extensions that promote virus transmission [32, 33]. During infection these viruses express a kinase, US3, which phosphorylates and activates PAK1 (p21-activated kinase Group I) to mediate changes in the actin cytoskeleton. In contrast, the alphavirus structural proteins do not contain a known kinase activity, so cytoskeletal remodeling is presumably triggered by other means. This could involve upstream effects on Rho-GTPases, which act to change the membrane-associated cytoskeleton and induce the formation of plasma membrane protrusions by stimulating actin polymerization [34]. Alternatively, the viral proteins may act on downstream effector molecules such as PAK1 [1, 32]. Given that inhibitors of these key proteins can affect both actin filaments and microtubules, and can also block delivery of viral envelope proteins to the plasma membrane [e.g., ref 31], inhibitors may be problematic for dissecting alphavirus extension formation. The ability of the viral structural proteins to induce extensions provides a relatively simple system to directly look for effects on critical cytoskeletal pathways.

In addition to cell-free virus infection, a number of viruses infect also or perhaps primarily via cell-to-cell transmission, with potential advantages in efficiency and in protection from neutralizing antibodies [1, 6]. A study of CHIKV infection showed that antibodies from the plasma of convalescing patients could neutralize free virus but that intercellular transmission in vitro was relatively resistant to neutralization [18]. It is unclear whether a similar mechanism occurs in vivo, but it is tempting to speculate that formation of the extensions we describe could help the virus escape neutralization and promote intercellular transmission. Enrichment of virus budding at the contact between infected and uninfected cells could provide a higher local multiplicity of infection to increase the chances of a successful infection, even when the receptor is down-regulated. Definition of the mechanism of extension formation will allow a more complete understanding of its importance during in vivo infection and identify potential targets for the inhibition of virus transmission.

## Methods

### Cells

BHK-21 cells (originally obtained from the lab of Dr. Ari Helenius) were maintained at 37°C in Dulbecco’s modified Eagle’s medium containing 5% fetal calf serum (FCS), 10% tryptose phosphate broth, 100 U penicillin/ml, and 100 μg streptomycin/ml. Vero cells (obtained from ATCC) were cultured at 37°C in Dulbecco’s modified Eagle’s medium containing 10% FCS, 100 U penicillin/ml, and 100 μg streptomycin/ml. Human Umbilical Vein Endothelial Cells (HUVEC) were obtained from Lonza and maintained in endothelial cell growth media BulletKit at 37°C (Lonza CC-3124). The CHO-K1 cell line and its derivative, the glycosaminoglycan-deficient CHO-745 cell line (both obtained from Dr. Jeff Esko) [21] were cultured at 37°C in alpha-minimal essential medium containing 10% FCS, 100 U penicillin/ml, and 100 μg of streptomycin/ml.

Vero cell lines stably expressing the PM-GFP marker were generated as previously described [12]. Vero cell lines stably expressing GFP-actin were generated using a previously described GFP-actin construct [35] kindly provided by Dr. Diane Cox at Albert Einstein College of Medicine. Briefly, in this construct GFP was fused to the N-terminus of the human actin protein and cloned into the pcDNA3.1 expression plasmid. Vero cells were transfected for 6 h with the GFP-actin plasmid using Lipofectamine 2000 according to the manufacturer’s instructions, cultured for 48 h in Vero medium, and then selected for 2 weeks in the presence of 1mg G418/ml (G418 from Sigma-Aldrich). Cells were then sorted to obtain single cell clones expressing medium/low levels of GFP-actin. Two independent clones were maintained for this study, and gave equivalent results.

### Viruses

Virus stocks were prepared from the SFV pSP6-SFV-4 infectious clone [36] and the SINV dsTE12Q infectious clone [37] by in vitro transcription of the virus RNA and electroporation of BHK-21 cells [36]. Electroporated cells were cultured for 12 to 18 h, and the media were collected and titered by plaque assay on BHK-21 cells. The Chikungunya virus vaccine strain 181/25 was propagated and titered in BHK-21 cells.

Labeled SINV viruses were generated from the dsTE12Q infectious clone as previously described [12]. In brief, E2 was labeled by insertion of either oxGFP (a cysteine-less variant of GFP)[38] or mCherry after the PE2 furin cleavage site to gernerate WT E2-GFP or WT E2-mCherry, respectively. The E2 Y400K mutation was generated by in vitro mutagenesis as previously described [12]. These labeled mutant viruses are here referred to as Y400K E2-GFP or Y400K E2-mCherry. The SFV E1 G91D and SFV E1 D188K mutants were generated by site-mutagenesis of the pSP6-SFV-4 infectious clone as previously described [23, 25], and WT SFV and mutant virus RNAs were produced by in vitro transcription of the respective pSP6-SFV-4 infectious clones.

### Antibodies

R6 mouse monoclonal (MAb) against SINV E2 [39] (kindly provided by William Klimstra) was used for immunofluorescence studies. The antibody used against SFV E2 was the MAb E2-1 [40]. A rabbit polyclonal antibody to the SFV envelope proteins [40] was used to detect E2 and E1 in SFV- and CHIKV-infected cells. The 6G7 anti-β-tubulin antibody developed by Willi Halfter and the 12G10 anti-α- tubulin developed by Frankel and Nelsen were obtained from the Developmental Studies Hybridoma Bank (developed under the auspices of NICHD and maintained by the University of Iowa, Department of Biology, Iowa City, IA). The anti-α-tubulin antibody (ab18251) was purchased from Abcam. AlexaFluor (AF)-conjugated phalloidin and AF 488-, 561-, or 405-conjugated anti-mouse or anti-rabbit antibodies were obtained from Molecular Probes.

### Virus infection

Vero cells were infected with viruses for 90 min at 37°C using the multiplicity of infection (MOI) indicated in the figure legends. Cells were then washed twice and incubated in fresh medium at 37°C for the indicated times. To analyze the budding-defective Y400K SINV mutant or the fusion-deficient SFV mutants G91D or D188K, Vero cells were transfected with WT or mutant viral RNA using Lipofectamine 2000 in Opti-MEM (Invitrogen) according to the manufacturer’s instructions and incubated for 6 h. Cells were then transferred to fresh culture medium and incubated for the total time described for each experiment. The time course of WT virus protein expression and particle release was similar using infection or transfection.

### Viral protein expression

The region encoding the structural proteins from the SFV infectious clone was inserted into pcDNA3.1 plasmid (construct provided by Chantal Chanel-Vos). This plasmid is indicated as WT+Cp plasmid and encodes Cp-E3-E2-6K-E1. It was used as a template to construct the following expression vectors (constructs provided by Gwen Taylor): WT-Cp encoding the glycoproteins and 6K but not the Cp; Y399R+Cp or Y399R-Cp encoding the structural proteins with or without Cp and the E2 Y399R mutation, which prevents E2-Cp interactions [11].

For expression studies Vero cells were transfected with these plasmids using Lipofectamine 2000 according to manufacturer’s instructions and incubated for 6 h at 37°C. Cells were then transferred to fresh culture medium and incubated for a total of 24h.

### Immunofluorescence microscopy and quantitation of extensions

Cells were cultured overnight on 8 well MatTek glass-bottom culture chambers (number 1.5; P35G-1.5-14-C; MatTek Corporation). HUVEC cells were cultured overnight on 8 well IBIDI microscopy plastic-bottom culture chambers (number 1.5; 8 well ibiTreat; IBIDI 80826). Cells were then transfected with viral RNAs or expression plasmids, or infected with virus stocks. At the time indicated in the legends for each experiment, cells were fixed with 4% paraformaldehyde (Electron Microscopy Science) for 10 min at room temperature and then permeabilized with 0.2% Triton X-100 for 7 min at room temperature. Cells were stained to detect viral and celluar proteins. Confocal images were acquired using an LSM5 Live DuoScan confocal microscope system (DuoScan microscope; Carl Zeiss MicroImaging, Inc.) with a 63X oil objective (numerical aperture 1.4).

For quantitation of extensions a total of 10 infected cells or cell pairs were counted in each of 3 independent experiments (total of 30 cells/cell pairs). Co-staining for E2 and actin was used to define actin-positive extensions in infected cells. Unless otherwise indicated in the legend, intercellular extensions were defined by the presence of actin, tubulin, and viral envelope proteins, and their origin from the infected cell to form a physical contact with a neighboring cell. In cases in which an intercellular extension formed branches that contact a target cell at 2 or more points, this was still counted as one extension. Measurements of the number of extensions were performed semi-automatically using Volocity software (version 6.2.1; PerkinElmer).

### Live cell microscopy, data acquisition, and image analysis

Vero cells stably expressing GFP-actin were grown on MatTek glass-bottom culture dishes and infected with WT-mCherry virus. At 6 h after infection, the medium was replaced with imaging medium (Dulbecco’s modified Eagle’s medium without phenol red or NaHCO3 [D-2902; Sigma] supplemented with 10mM HEPES, pH 7.0, 10% fetal bovine serum, 100 U penicillin/ml, and 100 g streptomycin/ml and glucose to a final concentration 3 g/l). Imaging chambers were sealed with Parafilm, and cells were maintained on a heated microscope stage at 37°C. For live Epi-fluorescence microscopy we used a modified commercial, objective-based TIRF Olympus IX71 microscope in the Gruss Lipper Biophotonics Center, Albert Einstein College of Medicine. The light sources of the Olympus IX71 microscope were replaced by five lasers, two of which were used in this work: 488-nm (IMA series;Melles Griot) and 561-nm (Jive 10; Cobolt) lasers. Rapid switching and shuttering of the lasers was accomplished with an acousto-optic tunable filter. The epifluorescence illumination mode was used for each laser line. A 60X oil objective (numerical aperture 1.45) was used, and sequential images were acquired every 60 s with a 50-ms exposure using a back-illuminated electron-multiplying chargecoupled-device camera (DU-897; Andor). Images were collected using Metamorph software (Molecular Devices, Sunnyvale, CA) and analyzed using Volocity three-dimensional image analysis software. Time series were generated using ImageJ software (National Institutes of Health, Bethesda, MD), and images for figures were processed with Adobe Photoshop software.

### Scanning electron microscopy

Vero cells were cultured on glass coverslips. Cells were mock infected or infected with WT SINV at an MOI of 5 and fixed after 9 h at 37°C. Alternatively, cells were mock transfected or transfected with the plasmids WT+Cp or Y399R+Cp and fixed after 24 h at 37°C. Fixation was for 20 min at room temperature with 4% paraformaldehyde, 2% glutaraldehyde in phosphate-buffered saline. Cells were then fixed with 2.5% glutaraldehyde, postfixed in 2% osmium tetroxide, dehydrated in ethanol, critical-point dried (Tousimis Samdri 790 critical point dryer), and coated with chromium (EMS 150T-ES sputtercoater). Cells were imaged using a Zeiss Supra 40 field emission scanning electron microscope (SEM) using a secondary electron detector.

### NRAMP dowregulation and SINV infection

Vero cells were pretreated by adding ammonium iron citrate (200 μg/ml) to the culture medium every day for 2 days, or mock-treated. Cells were then plated on MatTek glass-bottom culture dishes, grown overnight at 37°C in the presence or absence of ammonium iron citrate, and infected with SINV or SFV at a MOI of 5 for 2h at 37°C. The medium was then replaced with fresh medium with ammonium chloride to prevent secondary infection. Cells were incubated ON at 37°C, fixed, permeabilized, and stained with MAbs against SINV or SFV E2 to identify infected cells. Images were acquired using a 20X objective in an epifluorescence microscope. All the positive cells in the well were manually quantified and in each case the control condition was fixed as 1 while the treated condition was expressed relative to this value.

### Co-culture experiments

1.5x10^4^ Vero cells were plated in wells of MatTek glass-bottom culture dishes and cultured overnight at 37°C, to a density of ~3x10^4^ cells per well. The cells were then infected with SINV or SFV at an MOI of 5, or transfected with the viral RNA as detailed above. These cells will be referred to as producer cells. After 2 h at 37°C these cells were washed 3 times with phosphate-buffered saline and fresh medium was added. After a total of 5h of infection 3x10^4^ uninfected Vero PM-GFP cells were plated into the wells. These cells will be referred to as target cells. To quantitate formation of intercellular extensions, the cells were fixed and permeabilized at 8 hours post-infection, and stained for SINV or SFV E2. Images were acquired using a 63X objective in an epifluorescence microscope. To quantitate infection of target cells, the co-cultured cells were incubated for a total of 24 hours post-infection, fixed and permeabilized, and stained for SINV or SFV E2. Images were acquired using a 20X objective in an epifluorescence microscope. In each case, 10 random fields in each of 3 independent experiments were imaged and later quantitated. The infection of the PM-GFP target cells was compared to the total number of Vero PM-GFP cells.

To test the effect of NRAMP in the target cells, Vero PM-GFP cells were pretreated with iron for 2 days as detailed above and maintained in high iron conditions during the length of the co-culture experiment. To test the effect of NRAMP in the producer cells, Vero cells were pretreated with iron for 2 days and transfected with viral RNA for 5 h. The cells were then co-cultured with Vero PM-GFP target cells and maintained in high iron conditions during the length of the co-culture experiment. Controls were not pretreated or cultured in high iron. Quantitation of infection was performed as above. For each condition, infection of target cells from control, non-iron treated conditions was expressed as 1, and infection of the treated cells was compared to that of the control cells.

### Statistical analysis

The Microsoft Excel program was used to calculate statistical significance by a two-tailed unpaired Student’s t-test.

## Supporting Information

S1 Movie Movie showing formation of intercellular extensions by contact and migration of infected cells. Vero cells stably expressing GFP-actin were infected with WT-mCherry SINV and incubated for 7 h at 37°C. The cells were then imaged using the TIRF microscope in the wide-field mode and both the 561-nm and 488-nm lasers. Images were acquired every 10 s for 60 min. Bar = 10 μm.
(AVI)

S1 Fig Alphavirus structural proteins are present in the intercellular extensions. Vero cells were transfected with WT SINV or Y400K SINV RNA, incubated at 37°C for 8 h, and fixed. Cells were permeabilized and stained with monoclonal antibodies to detect the viral E2 envelope protein and capsid protein. Cells were imaged by confocal microscopy. Images from one optical section are shown and are representative of two independent experiments. Bar = 20 μm.
(TIF)

S2 Fig Intercellular extensions are induced by various alphaviruses in primary human cells. HUVECs were mock-infected (Uninf.), transfected with SINV Y400K RNA, or infected with WT SINV, SFV or CHIKV (MOI 20, 10, 10, respectively). Cells were then incubated at 37°C for 11 h, fixed and permeabilized, and stained with antibodies to detect viral envelope proteins (virus GP) and α-tubulin, and with phalloidin to detect F-actin. Cells were imaged by confocal microscopy. Images from one optical section are shown and are representative of three independent experiments. Bar = 20 μm.
(TIF)

S3 Fig Formation of intercellular extensions is independent of the presence of heparan sulfate. (A) WT CHO cells or CHO 745 mutant cells (glycosaminoglycan deficient) were transfected with SINV WT or Y400K RNA. Cells were then incubated at 37°C for 11 h, fixed and permeabilized, and stained with antibodies to detect the viral envelope protein E2 and α-tubulin, and with phalloidin to detect F-actin. Cells were imaged by confocal microscopy. Images from one optical section are shown and are representative of three independent experiments. Bar = 20 μm. (B) The number of intercellular extensions per infected cell (n = 10) was quantitated based on their positive staining for both actin and tubulin and their contact with a neighboring cell. Graph in B shows the mean and standard deviation of three independent experiments, with 10 cells quantitated in each sample. * P<0.05, ***P<0.001.
(TIF)

S4 Fig Intercellular extensions are not stabilized by frustrated phagocytosis. Vero cells were infected with WT-SINV (MOI = 10), incubated at 37°C for 9 h, and fixed. Cells were permeabilized and stained with antibodies to detect the viral E2 protein and the following phagocytosis/endocytosis markers: (A) caveolin 1 (Cav-1), (B) clathrin heavy chain (CHC), and (C) dynamin 2 (Dyn2). Images were acquired with the DuoScan confocal microscope and are representative of the images from two independent experiments. Merge of all the optical sections is shown. Bar = 20 μm. Insets at the right of the figures show the indicated endocytic marker staining in regions of the contact sites, digitally zoomed 2.5X. No enrichment of the markers was observed.
(TIF)

S5 Fig Virus cell-cell transmission to NRAMP2-depleted cells. Effect of NRAMP downregulation on infection of co-cultures. Vero cells were infected with SINV or SFV (MOI = 5) and incubated for 5 h at 37°C. Target Vero cells stably expressing the PM-GFP marker were cultured for 3 days in control media or media containing 200 μg/ml ammonium iron citrate to down-regulate the SINV receptor NRAMP2, and then plated onto the infected cells at an approximate ratio of 1:1. The co-cultures were then incubated for 19 h at 37°C in the continued presence of iron as indicated. The % infected cells was quantitated by staining with antibody to the SINV or SFV E2 protein. The graph represents the mean and standard deviation of three independent experiments, with infection normalized to that of control cells (which was set to 1).
(TIF)

S6 Fig Non-budding mutant is not transmitted to target cells. Vero cells were transfected with WT SINV or SINV Y400K mutant RNA and incubated at 37°C for 5 h (producer cells), and washed to remove RNA and transfection reagent (see methods). Target Vero cells stably expressing the PM-GFP marker were cultured for 3 days in control media or media containing 200 μg/ml ammonium iron citrate to down-regulate the SINV receptor NRAMP2, and then plated onto the transfected cells at an approximate ratio of 1:1. The co-cultures were then incubated for 19 h at 37°C in the continued presence of iron as indicated. The % of total target cells that was infected was quantitated by staining with antibody to the SINV E2 protein. The graph represents the mean and standard deviation of three independent experiments, with infection normalized to that of control cells (which was set to 1).
(TIF)

S7 Fig SINV can form microplaques in presence of neutralizing antibodies. (A) Neutralization of free virus by mAbs to SINV E2. SINV virus (1x10^5^ PFU) was incubated with control medium or medium containing SINV neutralizing antibodies at 37°C for 1 h. The mix was then added to a 24 well plate containing 1x10^5^ Vero cells and the cells incubated for 30h at 37°C. Cells were then fixed and permeabilized, and infection detected by immunofluorescent staining for the E2 glycoprotein. (B) Vero cells were incubated with SINV (MOI = 1) at 37°C for 2 h. The infection medium was then replaced with control medium (left column), medium containing 20 mM NH_4_Cl (center column), or medium containing neutralizing antibodies at the same concentration as panel A (right column). Cells were incubated at 37°C for a total of 30 h, fixed and permeabilized. The E2 protein was detected by immunofluorescent staining and the nuclei by Hoechst dye.
(TIF)
