# Supplementary figures and images for: Intercellular Extensions Are Induced by the Alphavirus Structural Proteins and Mediate Virus Transmission
Source: PLoS Pathog. 2016 Dec 15;12(12):e1006061. doi: 10.1371/journal.ppat.1006061 (PMC5158078; doi:10.1371/journal.ppat.1006061)

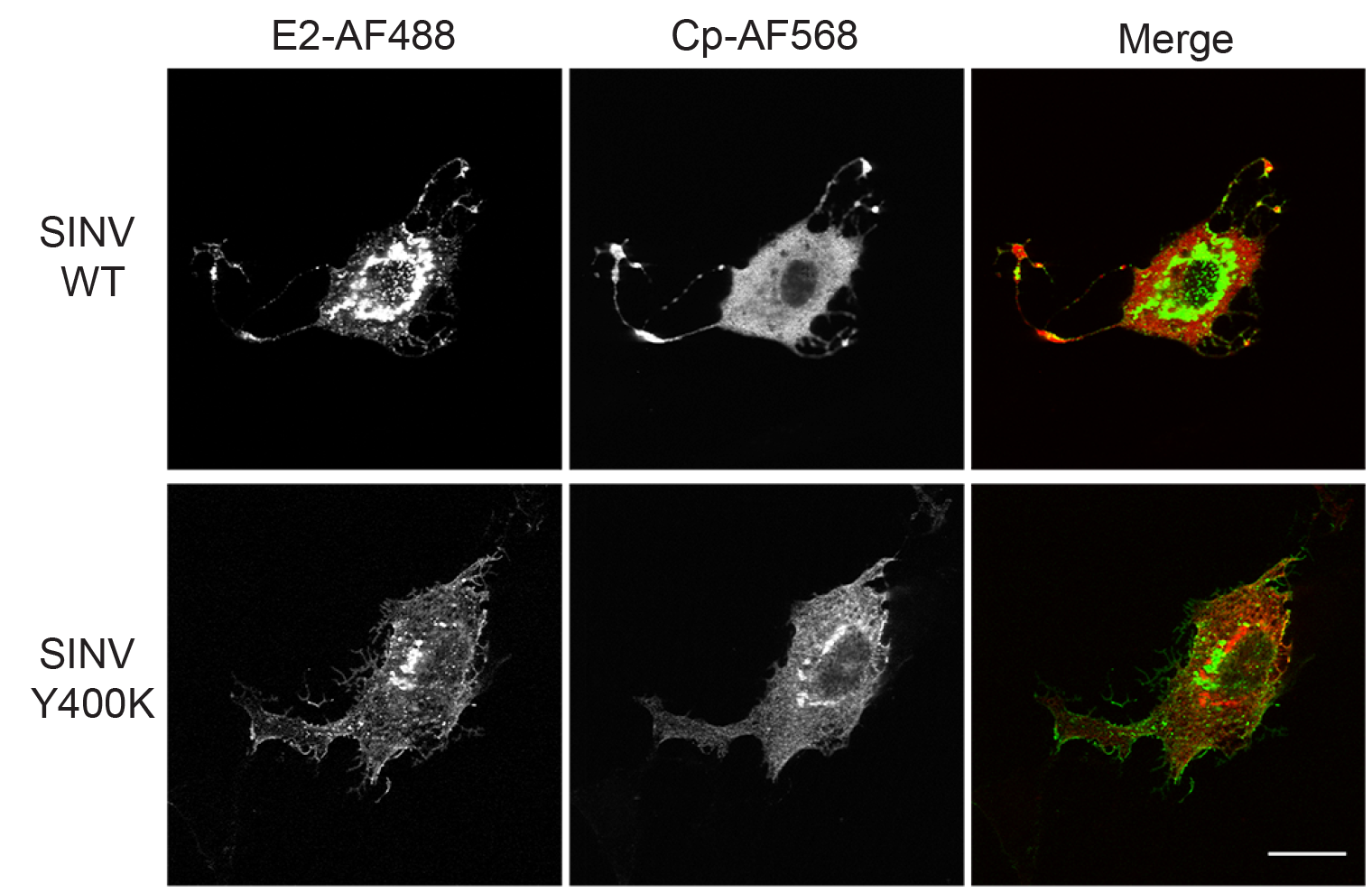

Supplement: S1 Fig — Vero cells were transfected with WT SINV or Y400K SINV RNA, incubated at 37°C for 8 h, and fixed. Cells were permeabilized and stained with monoclonal antibodies to detect the viral E2 envelope protein and capsid protein. Cells were imaged by confocal microscopy. Images from one optical section are shown and are representative of two independent experiments. Bar = 20 μm. (TIF) [file ppat.1006061.s002.tif]

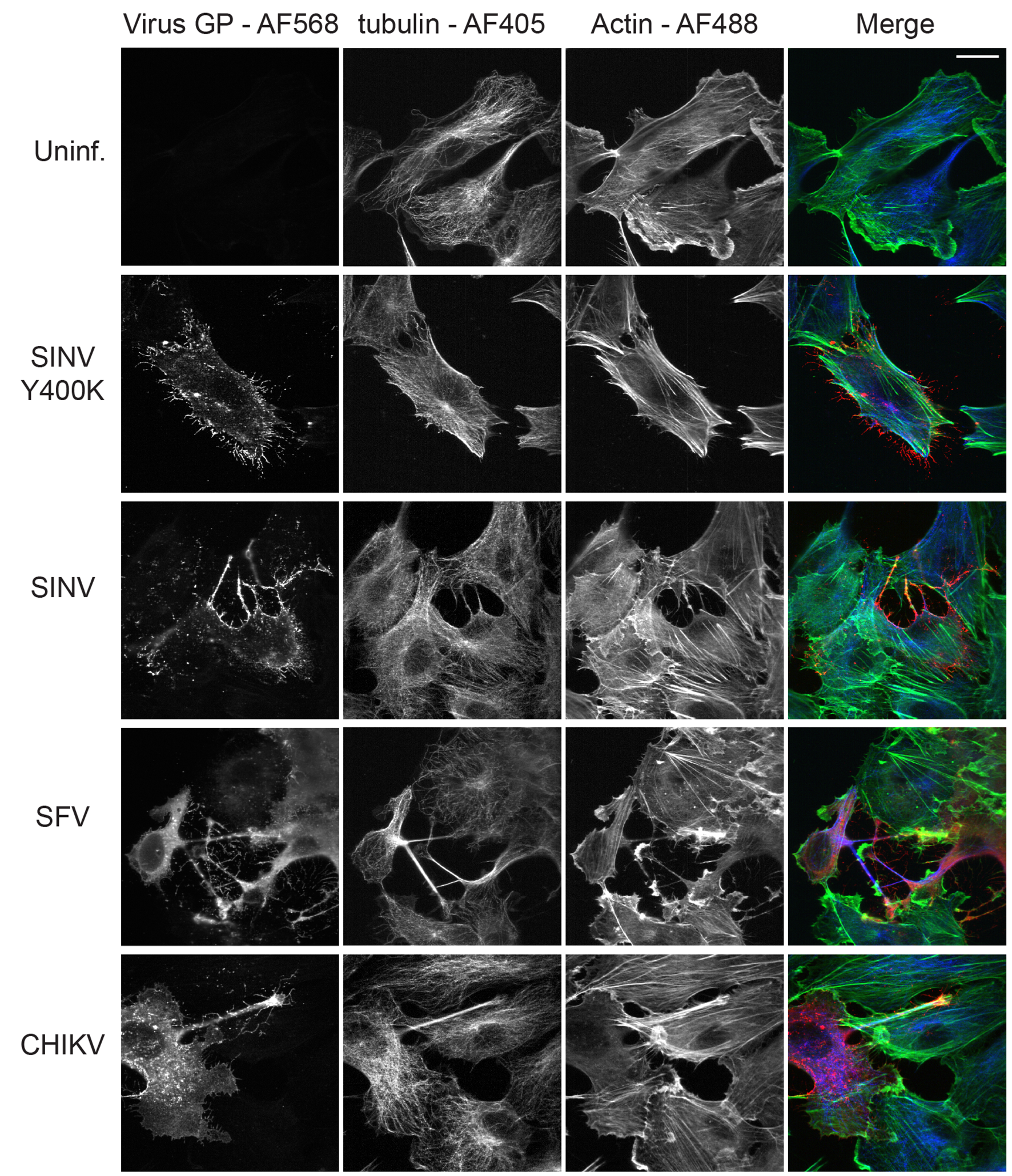

Supplement: S2 Fig — HUVECs were mock-infected (Uninf.), transfected with SINV Y400K RNA, or infected with WT SINV, SFV or CHIKV (MOI 20, 10, 10, respectively). Cells were then incubated at 37°C for 11 h, fixed and permeabilized, and stained with antibodies to detect viral envelope proteins (virus GP) and α-tubulin, and with phalloidin to detect F-actin. Cells were imaged by confocal microscopy. Images from one optical section are shown and are representative of three independent experiments. Bar = 20 μm. (TIF) [file ppat.1006061.s003.tif]

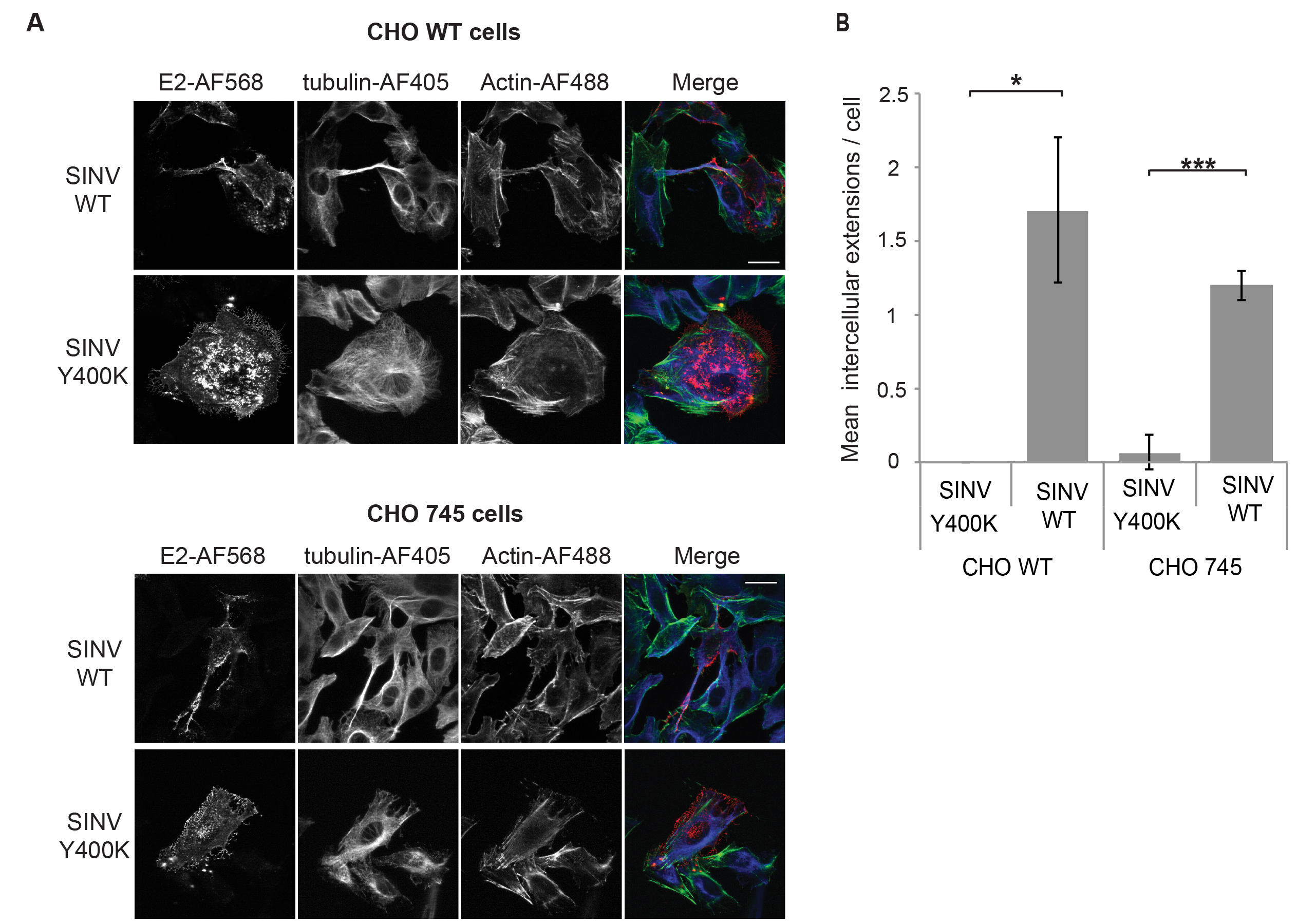

Supplement: S3 Fig — (A) WT CHO cells or CHO 745 mutant cells (glycosaminoglycan deficient) were transfected with SINV WT or Y400K RNA. Cells were then incubated at 37°C for 11 h, fixed and permeabilized, and stained with antibodies to detect the viral envelope protein E2 and α-tubulin, and with phalloidin to detect F-actin. Cells were imaged by confocal microscopy. Images from one optical section are shown and are representative of three independent experiments. Bar = 20 μm. (B) The number of intercellular extensions per infected cell (n = 10) was quantitated based on their positive staining for both actin and tubulin and their contact with a neighboring cell. Graph in B shows the mean and standard deviation of three independent experiments, with 10 cells quantitated in each sample. * P<0.05, ***P<0.001. (TIF) [file ppat.1006061.s004.tif]

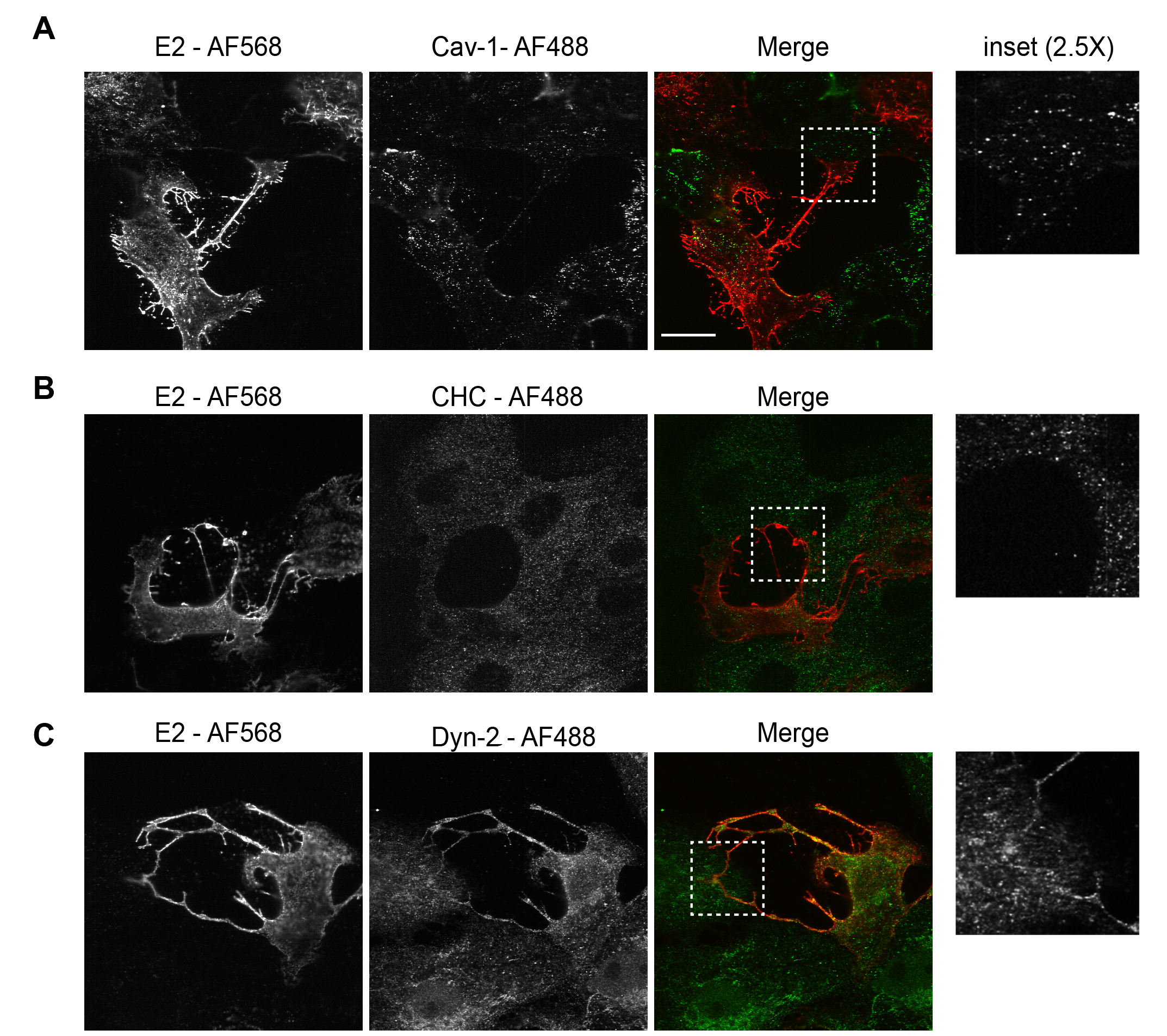

Supplement: S4 Fig — Vero cells were infected with WT-SINV (MOI = 10), incubated at 37°C for 9 h, and fixed. Cells were permeabilized and stained with antibodies to detect the viral E2 protein and the following phagocytosis/endocytosis markers: (A) caveolin 1 (Cav-1), (B) clathrin heavy chain (CHC), and (C) dynamin 2 (Dyn2). Images were acquired with the DuoScan confocal microscope and are representative of the images from two independent experiments. Merge of all the optical sections is shown. Bar = 20 μm. Insets at the right of the figures show the indicated endocytic marker staining in regions of the contact sites, digitally zoomed 2.5X. No enrichment of the markers was observed. (TIF) [file ppat.1006061.s005.tif]

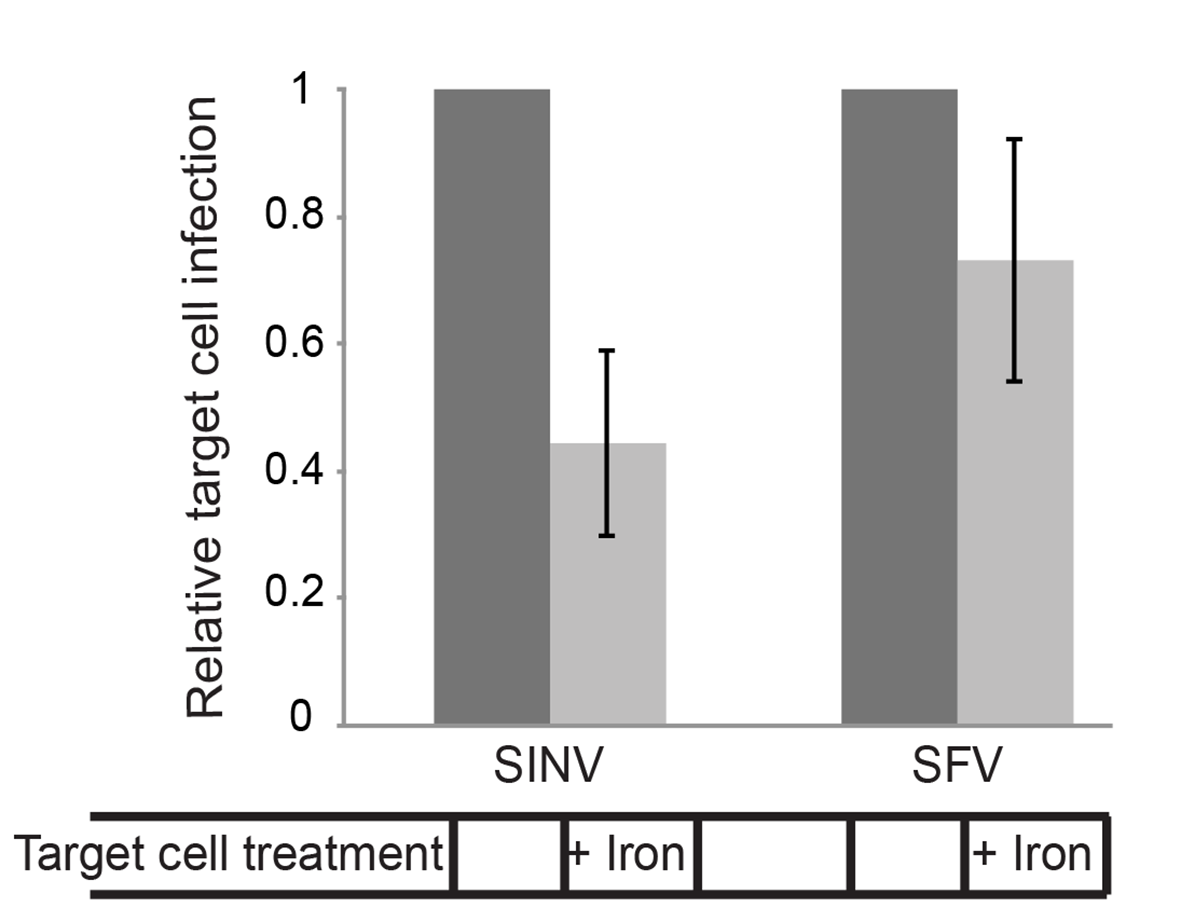

Supplement: S5 Fig — Effect of NRAMP downregulation on infection of co-cultures. Vero cells were infected with SINV or SFV (MOI = 5) and incubated for 5 h at 37°C. Target Vero cells stably expressing the PM-GFP marker were cultured for 3 days in control media or media containing 200 μg/ml ammonium iron citrate to down-regulate the SINV receptor NRAMP2, and then plated onto the infected cells at an approximate ratio of 1:1. The co-cultures were then incubated for 19 h at 37°C in the continued presence of iron as indicated. The % infected cells was quantitated by staining with antibody to the SINV or SFV E2 protein. The graph represents the mean and standard deviation of three independent experiments, with infection normalized to that of control cells (which was set to 1). (TIF) [file ppat.1006061.s006.tif]

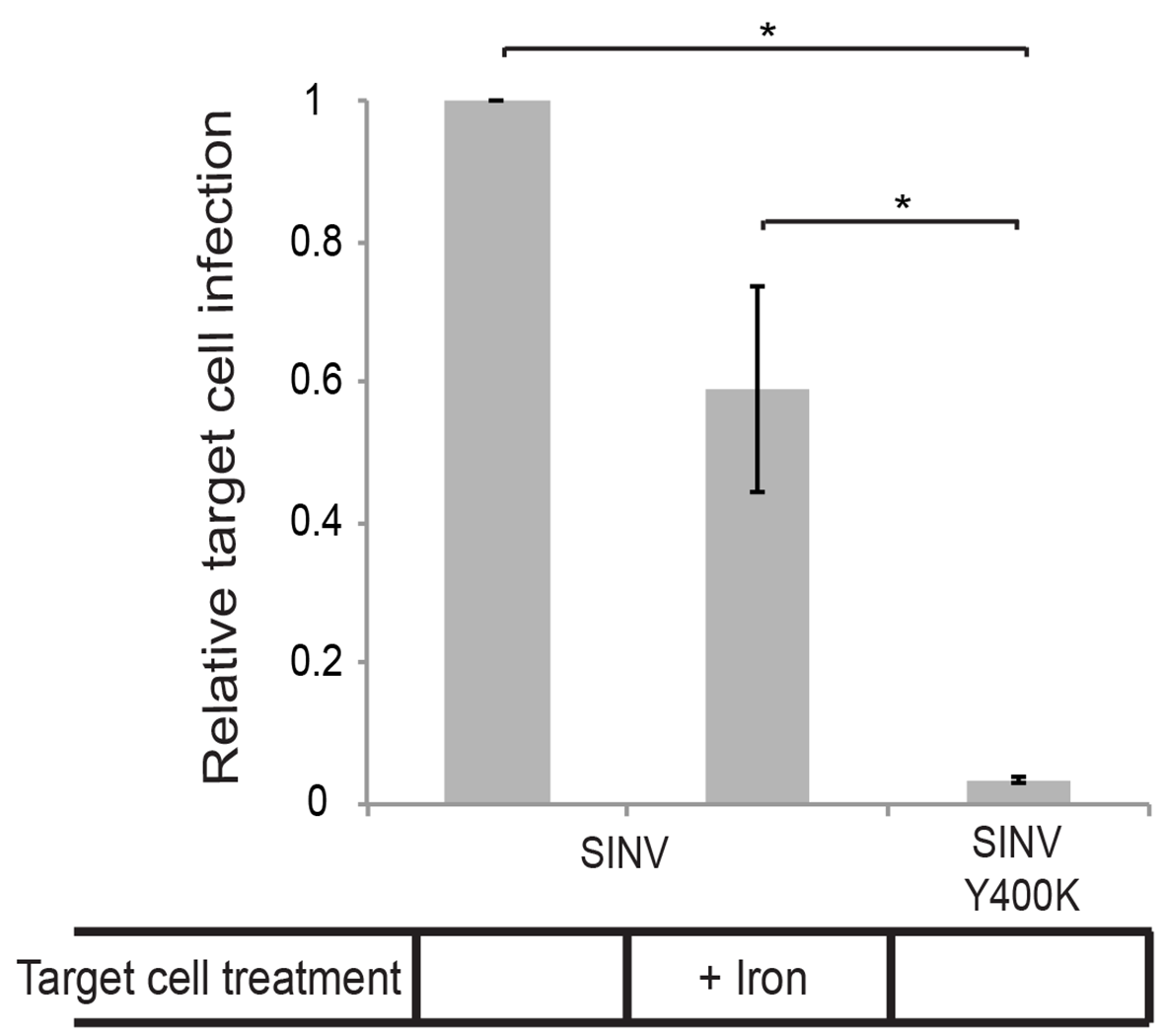

Supplement: S6 Fig — Vero cells were transfected with WT SINV or SINV Y400K mutant RNA and incubated at 37°C for 5 h (producer cells), and washed to remove RNA and transfection reagent (see methods). Target Vero cells stably expressing the PM-GFP marker were cultured for 3 days in control media or media containing 200 μg/ml ammonium iron citrate to down-regulate the SINV receptor NRAMP2, and then plated onto the transfected cells at an approximate ratio of 1:1. The co-cultures were then incubated for 19 h at 37°C in the continued presence of iron as indicated. The % of total target cells that was infected was quantitated by staining with antibody to the SINV E2 protein. The graph represents the mean and standard deviation of three independent experiments, with infection normalized to that of control cells (which was set to 1). (TIF) [file ppat.1006061.s007.tif]

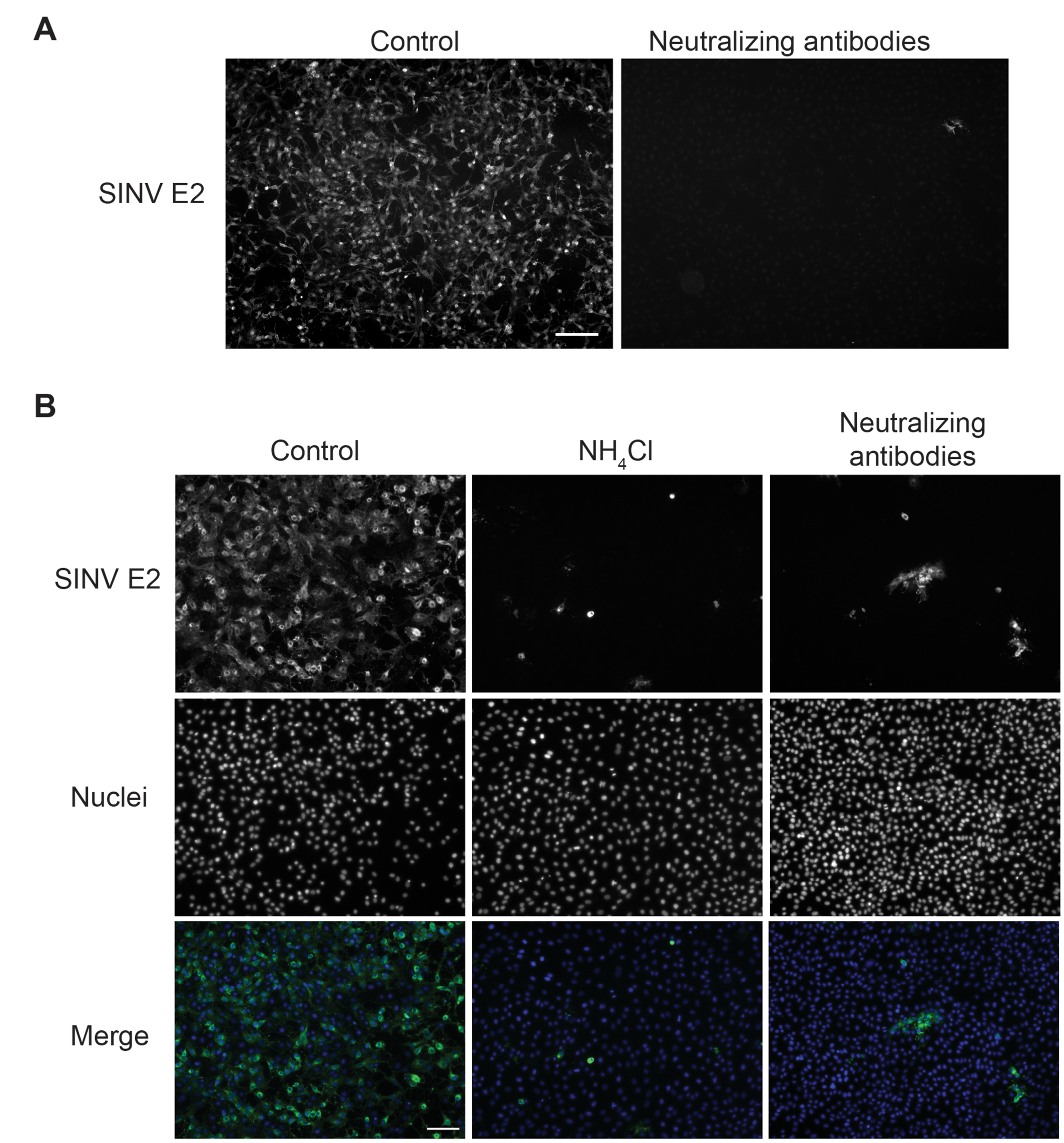

Supplement: S7 Fig — (A) Neutralization of free virus by mAbs to SINV E2. SINV virus (1x10^5^ PFU) was incubated with control medium or medium containing SINV neutralizing antibodies at 37°C for 1 h. The mix was then added to a 24 well plate containing 1x10^5^ Vero cells and the cells incubated for 30h at 37°C. Cells were then fixed and permeabilized, and infection detected by immunofluorescent staining for the E2 glycoprotein. (B) Vero cells were incubated with SINV (MOI = 1) at 37°C for 2 h. The infection medium was then replaced with control medium (left column), medium containing 20 mM NH4Cl (center column), or medium containing neutralizing antibodies at the same concentration as panel A (right column). Cells were incubated at 37°C for a total of 30 h, fixed and permeabilized. The E2 protein was detected by immunofluorescent staining and the nuclei by Hoechst dye. (TIF) [file ppat.1006061.s008.tif]
